# Unprofessional behaviour of GP residents and its remediation: a qualitative study among supervisors and faculty

**DOI:** 10.1186/s12875-021-01609-3

**Published:** 2021-12-20

**Authors:** Pieter C. Barnhoorn, Vera Nierkens, Marianne C. Mak-van der Vossen, Mattijs E. Numans, Walther N. K. A. van Mook, Anneke W. M. Kramer

**Affiliations:** 1 Department of Public Health and Primary Care, Leiden University Medical Center, Leiden, Hippocratespad 21, Zone V0-P, PO Box 9600, 2300 RC Leiden, The Netherlands; 2 Department of Public Health research institute, Amsterdam UMC, University of Amsterdam, Amsterdam, the Netherlands; 3 Department of Intensive Care Medicine and Academy for Postgraduate Medical Training, Maastricht University Medical Centre, Maastricht, the Netherlands; 4 School of Health Professions Education, Maastricht University, Maastricht, the Netherlands

**Keywords:** Professionalism lapses, Unprofessional behaviour, Professionalism, Professional identity formation, General practice, Residents, Internship and residency, Remediation, Medical education, Continuing medical education (CME)

## Abstract

**Background:**

Lapses in professionalism have profound negative effects on patients, health professionals, and society. The connection between unprofessional behaviour during training and later practice requires timely identification and remediation. However, appropriate language to describe unprofessional behaviour and its remediation during residency is lacking. Therefore, this exploratory study aims to investigate which behaviours of GP residents are considered unprofessional according to supervisors and faculty, and how remediation is applied.

**Methods:**

We conducted eight semi-structured focus group interviews with 55 broadly selected supervisors from four Dutch GP training institutes. In addition, we conducted individual semi-structured interviews with eight designated professionalism faculty members**.** Interview recordings were transcribed verbatim. Data were coded in two consecutive steps: preliminary inductive coding was followed by secondary deductive coding using the descriptors from the recently developed ‘Four I’s’ model for describing unprofessional behaviours as sensitising concepts.

**Results:**

Despite the differences in participants’ professional positions, we identified a shared conceptualisation in pinpointing and assessing unprofessional behaviour. Both groups described multiple unprofessional behaviours, which could be successfully mapped to the descriptors and categories of the Four I’s model. Behaviours in the categories ‘Involvement’ and ‘Interaction’ were assessed as mild and received informal, pedagogical feedback. Behaviours in the categories ‘Introspection’ and ‘Integrity’, were seen as very alarming and received strict remediation. We identified two new groups of behaviours; ‘Nervous exhaustion complaints’ and ‘Nine-to-five mentality’, needing to be added to the Four I’s model. The diagnostic phase of unprofessional behaviour usually started with the supervisor getting a ‘sense of alarm’, which was described as either a ‘gut feeling’, ‘a loss of enthusiasm for teaching’ or ‘fuss surrounding the resident’. This sense of alarm triggered the remediation phase. However, the diagnostic and remediation phases did not appear consecutive or distinct, but rather intertwined.

**Conclusions:**

The processes of identification and remediation of unprofessional behaviour in residents appeared to be intertwined. Identification of behaviours related to lack of introspection or integrity were perceived as the most important to remediate. The results of this research provide supervisors and faculty with an appropriate language to describe unprofessional behaviours among residents, which can facilitate timely identification and remediation.

## Background

Unprofessional behaviour by physicians compromises patient-physician relationships, patient safety, and quality of care. Consequently, it can harm patients’ trust in the medical profession [1–6]. Professional behaviour – defined as “placing the best interests of patients at the center of everything you do” [7, 8] - on the other hand, positively affects patient-physician relationships, patient and physician satisfaction, as well as healthcare outcomes [9, 10].

Although lapses in professionalism are a part of learning [11, 12], research has also revealed a clear association between unprofessional behaviour during undergraduate and postgraduate training and unprofessional behaviour in later practice [13–17]. The positive approach of teaching and assessing professionalism and supporting Professional Identity Formation (PIF) is increasingly receiving attention [7, 18–20]. Nonetheless, the complementary approach of identifying and remediating unprofessional behaviour remains indispensable [7, 21]. Since unprofessional behaviour is a valuable signal indicating the stagnation of PIF, remediation is defined as “the process of facilitating corrections for physicians and trainees who are not on course to competence or a professional identity” [7, 22].. However, timely and adequate remediation of lapses in professionalism can only occur if these lapses are indeed identified as such [11, 22].

One important reason why lapses in professionalism are not identified or are identified too late, (often termed as ‘failure to fail’), is a lack of appropriate language to describe unprofessional behaviour and its remediation [12, 23]. This ‘failure to fail’ implicitly promotes the idea that unprofessional behaviour is acceptable. To overcome this problem, the Four I’s model for describing unprofessional behaviours was recently developed. The Four I’s model consists of descriptors for unprofessional behaviour, classified into four distinct categories; lack of Involvement (failure to engage) Integrity (dishonest behaviour); Interaction (disrespectful behaviour); and Introspection (poor self-awareness) [12, 24]. The descriptors and the four categories guide educators in how to document unprofessional behaviour, and in doing so, provide directions for effective remediation.

The Four I’s model originated from research in undergraduate medical education (UGME). Thorough research into unprofessional behaviour and its remediation in the postgraduate medical education (PGME) setting, including the application of the Four I’s model, is thus far absent [25, 26]. Nevertheless, PGME is a crucial period during which to support PIF, as residency remains the forge that moulds and tempers the physician-to-be. Therefore, understanding the development of professional behaviour and the way in which that behaviour is supported is needed to detect improvements [21, 27, 28]. Clear descriptors can assist in the timely identification of unprofessional behaviours observed in residents. Insight into the remediation process can identify efficacious remediation strategies and foster a culture in which residents’ needs for extra guidance regarding their PIF are acknowledged. The multi-level professionalism framework for remediation is one of the few tools to guide and facilitate remediation of unprofessional behaviour. It was developed to encourage reflection on six levels influencing professionalism: [1] the environment in which the learner functions, [2] the displayed behaviour, [3] the potential for behaviour or competencies, [4] the conceptions or convictions the learner holds true regarding the medical profession and his or her place in it, i.e. beliefs & values, [5] the way one defines oneself, or one’s identity, and at the model’s centre, [6] mission, or what drives people [7].

General practice (GP) is a unique context in which to address this issue. In primary care, the physician is the first port of call for patients entrusted to them and, consequently, this is where the foundation for patient-physician relationships, patient safety, quality of care and patients’ trust in the medical profession is laid.

Therefore, this exploratory study aims to:

1. investigate which behaviours of GP residents are considered unprofessional according to their GP clinical supervisors and faculty,
2. whether the Four I’s model may be appropriate to describe these behaviours of GP residents and
3. how remediation of unprofessional behaviour is applied.

## Methods

### Study design

Based on a constructivist paradigm, we conducted a qualitative study applying thematic analysis of data derived from:

1. focus group interviews with GP clinical supervisors
2. individual interviews with designated professionalism faculty members [29–31].

We chose to collect data from GP clinical supervisors and designated professionalism faculty members as both are confronted with unprofessional behaviours of residents, however each with different roles in the processes of identification and remediation. We chose to interview the designated professionalism faculty members individually since this approach facilitated retrieving detailed, yet potential confidential information. The constructivist paradigm of the study means that participants and researchers co-created the outcome of this study. The authors form an interdisciplinary research group of educational researchers, medical educators and doctors. They all have expertise in medical education research and in the field of medicine. VN is a health scientist specialised in health behaviour. The other authors are GPs, except for WvM who is an intensivist. A sixth-year medical student (MS) acted as observer and independent analyser in the interviews with faculty. We applied the consolidated criteria for reporting qualitative research (COREQ) [32].

### Study context

Before enrolling in a Dutch specialist GP training program, most recently graduated doctors work for some years in their field of interest to gain additional experience as a practicing physician. GP resideny training is offered at one of the eight Dutch GP training institutes and consists of three years of workplace-based learning, combined with formal training activities in a university setting. In years one and three of the program, GP-trainees work in a general practice where they are coached and instructed by a supervising senior GP. GP clinical supervisors are offered faculty development programmes in supervising and assessing GP-trainees. These compulsory programmes, in which the trainers are GP faculty and behavioural science teachers, are held multiple times annually at the affililted GP training institute. Year two of GP residency training consists of rotations in hospitals, nursing homes and psychiatric outpatient clinics with various supervisors. Trainees typically work four days a week in their training practice. On the fifth day, they participate in a so-called ‘day release program’ staffed by GP faculty and behavioural science teachers. On these days - designed to facilitate and deepen learning from experiences in practice - residents learn in small groups (10 to 15 residents) about case histories, protocols, skills and Entrustable Professional Activities (EPAs), with dedicated time for collaborative reflection. Residents’ progress towards standard performance is monitored using the proven reliable and valid Competency Assessment List (Compass), of which professionalism is an integral part [33]. Each of the eight Dutch GP training institutes has one designated professionalism faculty member – either a GP or behavioural scientist - responsible for attending to lapses in professionalism and remediation of unprofessional behaviour.

### Participants and procedure

At four training institutes across the Netherlands we asked a contact person responsible for the training of GP clinical supervisors to select existing training groups of GP clinical supervisors to participate in focus group interviews. We included four groups from Leiden (LUMC); one from Rotterdam (ErasmusMC); one from Maastricht (MaastrichtUMC+); and two from Groningen (UMCG). Hence, we aimed for a broad sampling regarding practice location (urban versus rural context) as well as supervisor ethnicity, gender and seniority [32, 34]. For the interviews with faculty, we invited all eight designated professionalism faculty members.

The main researcher (PB) conducted all focus group and individual interviews using a standardised interview guide. The semi-structured interviewguide (see Appendix) was based on a topic list derived from prevailing literature and pilot interviews with both GP clinical supervisors and faculty who did not participate in the main study. The main, open questions explored which behaviours the participants perceived as unprofessional and how remediation of these behaviours was undertaken. To facilitate openmindedness and to facilitate interplay among participants, the terms ‘professionalism’ and ‘remediation’ were not predefined. Before initiating each interview, a brief explanation of the aim of the study was provided. An observer was present to observe the interactions between interviewer and interviewees and between the participants, to better understand social norms and values of the topic. When needed, the observer asked questions to clarify and to drill down further in the detail. All interview recordings were transcribed verbatim.

### Data analysis

Data analysis began as soon as the first data were gathered. PB, VN and MS independently performed coding and analysis of the individual interviews and focus group interviews separately.

Because unprofessional behaviours among GP residents as well as the remediation strategies were not described previously and because we wanted to be sure to include all types of behaviour and strategies, we started with open, inductive coding. The process of analysis started with PB, VN and MS familiarising themselves with the data and providing line-by-line open coding, using a constant comparative approach to jointly develop codes and themes. To avoid individual biases, we took an interactive and reflective approach throughout the cyclic process of data gathering, coding and analysis [35, 36]. PB, VN and MS developed an initial preliminary codebook, which was discussed within the team.

Coding and analysing in an interdisciplinary team facilitated the creation of unique insights from diverging perspectives and multiple interpretations of the data. Differences in interpretation were discussed in regular team meetings with special attention to divergent codes. On key themes, consensus with the entire team was sought and found.

After completing this first round of open coding, and through comparison of the coding results to the relevant literature, the descriptors from the Four I’s model appeared suitable for describing the aforementioned unprofessional behaviours. The professionalism framework appeared suitable to analyse on which level remediation took place. Therefore, in a new round of coding, we adopted a deductive approach. Concerning unprofessional behaviours we used the descriptors from the Four I’s model as sensitising concepts and paid special attention to text fragments that did possibly not fit in the descriptors of this model [12, 24]. Concerning remediation we used the professionalism framework to differentiate what remediaton level was involved in the remediation when participants were confronted with professional behaviour.

## Results

Eight focus group interviews with 55 GP clinical supervisors (27 female, 28 male) from four different Dutch GP training institutes in Leiden (LUMC), Rotterdam (ErasmusMC), Maastricht (MaastrichtUMC+), and Groningen (UMCG) were held. Group sizes ranged from four to nine participants, with a mean of 7 participants. Designated professionalism faculty members responsible for attending to lapses in professionalism from all eight Dutch GP training institutes participated in the individual interviews (4 female, 4 male; 5 GPs, 3 psychologists). The interviews were held between summer 2018 and fall 2019.

During the focus group interviews, the GP clinical supervisors discussed what they perceived as unprofessional behaviours and the likelihood that they could address and correct this behaviour. The designated professionalism faculty members painted a similar picture regarding what they viewed as unprofessional behaviour. However, designated professionalism faculty members were confronted with more egregious behaviours, as milder unprofessional behaviours had already been remediated by the GP clinical supervisors. An impression of the discussions is given below. Illustrative quotes are used where appropriate and their source coded to denote; supervisor/faculty (S/F); gender (M/F); and interview number (n). First, GP residents’ behaviours that were considered unprofessional are described, then the remediation process that followed.

## Unprofessional behaviours

Despite the differences in participants’ formal positions (GP clinical supervisor or designated professionalism faculty member), we identified a shared conceptualisation in pinpointing and assessing unprofessional behaviour. During the first round of open coding of the data it became clear that most unprofessional behaviours paralleled the descriptors of the Four I’s model. In the subsequent deductive coding round, we were able to map all initial codes to the categories of the Four I’s model; Involvement; Integrity; Interaction; and Introspection.

When asked which behaviours of GP residents they considered ‘unprofessional’, most GP clinical supervisors and faculty started with the umbrella term ‘being unteachable’. Asked to elaborate on this term, they referred to residents who were unable to reflect on their own behaviour and adjust it accordingly. They listed behaviours including blaming external factors when receiving critical feedback, not accepting feedback, resisting change, not being aware of own limitations, or acting beyond their level of competence (see Table 1.). According to the participants, these behaviours all illustrated a lack of *introspection*. GP clinical supervisors and faculty assessed behaviour from this category as very problematic, since they viewed introspection as indispensable for change. Therefore, they were very outspoken and decisive in their judgement when they suspected a lack of introspection.

**Table 1:** Twenty-four descriptors for unprofessional behaviour observed in GP residents, mapped to the Four I’s model

Involvement	Integrity
Absent or late for assigned activities^3^	Lying^3^
Poor reliability^3^	Acting without required consent^2^
Poor responsibility^3^	
Poor availability^3^	
Lack of conscientiousness^3^	
Tardiness^1^	
Cutting corners^2^	
Minimal acceptable level of performance^3^	
Poor teamwork^1^	
Not meeting deadlines^1^	
Language difficulties^1^	
Interaction	Introspection
Poor verbal/non-verbal communication^2^	Blaming external factors rather than own inadequacies^1^
Discrimination^2^	Not accepting feedback^1^
Showing a lack of empathy^1^	Resisting change^1^
Overly informal behaviour^2^	Not being aware of own limitations^1^
	Acting beyond own level of competence^1^
	Nervous exhaustion complaints^3^
	Nine-to-five mentality^1^

behaviours mentioned by both groups, GP clinical supervisors alone or faculty alone are indicated with superscripts ^1^, ^2^ and ^3^

*‘People who actually have no introspection or cannot show it, you shouldn’t let them enter the GP profession, because they will end up unhappy. That’s neither good for future patients nor for the residents themselves.’* SM6

*‘They of course receive feedback like, “What you are doing now is incorrect, do it this way.” Then they will try to learn the ‘trick’ and they often succeed quite well, but if the setting is different, the ‘trick’ won’t work. The feedback is not internalised. Explaining to this kind of residents why they cannot become a GP, is the most difficult.’* FF4

We found two new behaviours that did not match the descriptors of the Four I’s model: ‘Nervous exhaustion complaints’ and ‘Nine-to-five mentality’. Nervous exhaustion complaints are often referred to by GP clinical supervisors as ‘overload complaints’. These include behaviours like burn-out-related symptoms, e.g. being insecure or anxious, and unclear conditions that cause residents to drop out for a shorter or longer period of time.

According to both GP clinical supervisors and faculty, those behaviours illustrated a lack of introspection, since they illustrated that residents failed to acknowledge their poor fit to practice within the GP community. During their discussions about why they regarded this as unprofessional behaviour, GP clinical supervisors came to the conclusion that apparently the GP community has implicit expectations of what it takes to be a GP. Residents do not always notice these implicit expectations, as illustrated by the following focus group discussion between GP clinical supervisors:

*‘We have implicit expectations … implicit expectations of what it takes to be a GP. And if you do not meet these expectations, you are not entirely suited for the profession.’* SM2 … *‘This also has to do with norms.’* SM2 *‘For example, being ill and then sending an app the next day: 'I will use this day to recover.'* SF2 *‘I am never ill. What kind of nonsense is that?’* SM2 *‘That’s the younger generation mentality’.* SM2

Behaviours illustrating a lack of integrity, like lying and acting without required consent (see Table 1), were also seen as very problematic, but were rarely seen. Besides these serious unprofessional behaviours, participants mentioned behaviours illustrating a lack of involvement or interaction that caused less concern, but which also needed to be addressed and corrected. Both GP clinical supervisors and faculty gave examples of behaviours which illustrated a lack of involvement or interaction.

The first category of behaviours considered to be of lesser concern was a lack of involvement. This was described as a failure to engage, or inadequate handling of one’s tasks. GP clinical upervisors and faculty cited many examples from this category, for example, being absent or late for assigned activities, minimal acceptable level of performance and not meeting deadlines (see Table 1). However, they assessed these behaviours as being relatively unimportant. They even felt reluctant to qualify these behaviours as unprofessional, given the high probability that they could be corrected.

*‘You know, those smaller things like not being on time or being a bit shy and therefore difficult in interaction, or time management, that’s also part of it ... those are all things that we discuss, but then, at least, there are no doubts whether they* can *do it.’* FM1

Behaviours illustrating a lack of interaction occurred more often, but were still rare. Behaviours in this category included poor verbal/non-verbal communication, discrimination, showing a lack of empathy and overly informal behaviour (see Table 1). Behaviours illustrating a lack of involvement or interaction were primarily viewed as problematic only when combined with a lack of introspection. But, when lack of involvement or interaction appeared in isolation, both GP clinical supervisors and faculty assessed them as teaching opportunities and considered them part of the resident’s regular learning process.

*‘Professionalism, of course is also a learning point. So, in that sense you don't have to qualify behaviour as unprofessional right away. It is also a competence. So, you can grow into it, you can also learn things. Then it doesn't have to be qualified as unprofessional immediately.’* SF1

The focus group interviews with GP clinical supervisors yielded 29 different descriptions of unprofessional behaviours, which were coded into 17 different descriptors. The interviews with faculty yielded 34 different descriptions, which were coded into 21 different descriptors. Both groups of descriptors largely overlapped, resulting in 24 unique descriptors of unprofessional behaviours. Through discussions within the research team, we concluded that all descriptors expect for two (‘Nervous exhaustion complaints’ and ‘Nine-to-five mentality’), almost seamlessly fitted the original descriptors of the UGME Four I’s model. (Table 1).

Insight into how descriptors were derived from the data, using examples of the two new descriptors that were added are shown in Table 2.

**Table 2:** Examples of how descriptors were derived from the data

Quote	Description	Descriptor
… being unable to persist the practice of the profession [which is reflected in being] insecure, anxious, and in the end burn-out. FF4	Burn-out-related symptoms	Nervous exhaustion complaints
… illness after illness and then a pregnancy, with the result that after 2.5 years she still had not completed her first year of GP internship. FF6	Unclear conditions	
… she immediately puts her agenda on the table and says: “I want this… I have to pick up the children, so I have to leave at five. And I want this. And I am entitled to compensation” ... SM6	Work according to collective labour agreements mentality	Nine-to-five mentality
I had a resident who was very much a collective labour agreements resident. He wouldn’t do anything extra. He was in the training practice only for a short time, because then he would be off to a festival again … That’s a generational thing, I think. SM2	Work according to collective labour agreements mentality	
	Younger generation mentality	

## Remediation

For both GP clinical supervisors and designated professionalism faculty members, it appeared difficult to separate the diagnostic phase, in which the participants try to explore and understand the unprofessional behaviour, from the remediation phase. They described how exploring the unprofessional behaviour in a conversation with the resident often already had a remediating effect. And vice versa: it is not uncommon that the way in which residents cope with the remediation phase gives new input for the diagnostic phase.

GP clinical supervisors commented that they use the same tools to come to a diagnosis of unprofessional behaviour as GPs use in their diagnostic reasoning. Most GP clinical supervisors described the identification of unprofessional behaviour as a process that starts with getting a ‘sense of alarm’. We distinguished three types of senses of alarm. The first type is described as a ‘gut feeling’. GP clinical supervisors equate this type of alarm with the gut feeling they sometimes experience in diagnostic reasoning. There seems to be something wrong, but one cannot say exactly what it is.

*‘It is just your basic GP-skills… It’s the same as with your patients, it’s a gut feeling. Really the same feeling you have with these residents.’* SM4

The second sense of alarm is described as ‘a loss of enthusiasm for teaching’. GP clinical supervisors who experienced these feelings described the resident as being different from previous trainees they had supervised. Guiding such a resident cost a lot more energy, resulting in GP clinical supervisors questioning their commitment to the training of residents in general.

*‘But if it doesn't go very smoothly, then the whole point of the enthusiasm I had in the beginning is gone ... I had four trouble-free residents with whom I had a great relationship, I could talk to them and discuss difficult issues, you name it. But, when that’s not the case then I think: how much do I even like this?*’ SM1

The third type of alarm can be described as ‘fuss surrounding the resident’. Sometimes, other stakeholders working closely with the resident complain about them to the GP clinical supervisor.

*‘People at the pharmacy were grumbling at him. The psychologist at our practice said: "I hear stories about that guy." Our assistants also told us: “I get stories about that guy”. It really came from all sides.’* SF4

When the GP clinical supervisor has become fully aware of the sense of alarm, the diagnostic and remediation phases appeared to be intertwined. What follows varies from an informal ‘cup of coffee conversation’ to formally planned meetings. Further exploration and understanding, as well as assigning and assessing tasks often go hand in hand.

*‘You need to schedule a lot of meetings. A lot of writing. So, it really is a lot of work. … Then you can show what’s wrong. It doesn't make sense here and it doesn't make sense there.*’ SF4

This approach to remediation has a rather informal character until GP clinical supervisors discover that the remediation process goes beyond the usual supervision and coaching. When informal or ‘pedagogical’ feedback did not improve the situation, most GP clinical supervisors suspected a lack of introspection as the underlying problem. In these cases, the GP clinical supervisor made more explicitly use of the Compass – to exactly pinpoint and assess on which competency domain the resident was underperforming - and consulted faculty of the day release program to collaborate on a remediation plan. When lapses in professionalism persisted, the designated professionalism faculty member was consulted.

Unlike residents showing unprofessional behaviour in the categories Integrity, Involvement and Interaction, residents showing unprofessional behaviour in the Introspection category often seemed resistant to remediation on deeper levels than just behaviour, namely beliefs, values and identity. When introspection falls short and official remediation is needed, GP clinical supervisor and faculty seem to feel compelled to tighten the reins and remediate mainly on the more quantifiable level of behaviour and competencies, as they feel introspection to be a prerequisite for addressing deeper issues.

*‘In conversations together with faculty from the institute, we simply set a certain threshold: you really have to be able to do this, for example, in order to be able to be a GP.*’ SM1

Along the way from informal to formal feedback and from ‘pedagogical’ to stricter feedback, both GP clinical supervisors and faculty feel that their role changes from that of a supportive coach, which they enjoy, to a gatekeeper of the profession or even a police officer, which they do not enjoy at all.

## Discussion

### Principal findings

In this study we found that GP clinical supervisors and designated professionalism faculty members have closely corresponding conceptualisations regarding unprofessional behaviour among residents, and its remediation. Furthermore, most unprofessional behaviours paralleled the descriptors of the original Four I’s model and could be mapped to the categories of the Four I’s model. When GP clinical supervisors were confronted with unprofessional behaviour in residents, the diagnostic and remediation phases appeared to be non-consecutive, and intertwined. GP clinical supervisors and faculty considered behaviours from the categories Involvement and Interaction as needing an informal or ‘pedagogical‘type of remediation. However, if lack of Introspection or Integrity were assumed to be the underlying problem (both of which were seen as very alarming), then both GP clinical supervisors and faculty felt compelled to move to strict remediation on the more quantifiable levels of behaviour and competencies.

### Comparison with existing literature

#### Unprofessional behaviours

When GP clinical supervisors and faculty are confronted with unprofessional behaviour in residents, they use descriptions of unprofessional behaviours which to a large extent correspond to the descriptors outlined in the Four I’s model [12, 24].

This Four I’s model originated in the UGME setting. The work we have carried out confirms the continued value and validity of this model and indicates that only two new descriptors need to be added to adapt the original model to a PGME setting. Our results expand upon the Four I’s model by adding the descriptors: ‘Nervous exhaustion complaints’ and ‘Nine-to-five mentality’. These two novel descriptors reflect earlier literature findings that the current generation of physicians makes different behavioural choices compared to their older colleagues when certain values are at stake. This is especially the case in the postgraduate setting. In this phase of a physician’s career, work-life interference can be experienced as especially demanding. As a reaction, focus can shift from other to self [37–39]. The descriptors and their categorisation in the Four I’s (Involvement, Integrity, Interaction and Introspection and), can facilitate timely identification of this behaviour and in that way reduce the problem of ‘failure to fail’ [12, 23].

Our study further expands on the original Four I’s model by adding difference in assessment when confronted with different I’s. Behaviours from the categories Involvement and Interaction are seen as mild and as requiring informal pedagogical feedback. In contrast, behaviours from the Introspection and the Integrity categories are seen as very alarming and as requiring strict remediation. This difference is in line with the literature, as many earlier studies in medical education underline introspection as an absolute prerequisite for sustainable change [12, 19, 40–43]. Furthermore, the reluctance of GP clinical supervisors and faculty to qualify behaviours in the categories Involvement and Interaction as unprofessional, their emphasis on ‘professionalism being a competence in which growth can occur’ and their informal pedagogical approach to behaviour in these two categories is in line with the literature on PIF [19, 44, 45]. Because reflectiveness is a prerequisite for change, GP clinical supervisors halt their development-oriented PIF approach when confronted with behaviour in the Introspection category and ask for expert faculty’s help in further remediation [40, 42].

#### The interweaving of diagnosis and remediation

The tools used by both GP clinical supervisors and designated professionalism faculty members to identify unprofessional behaviours are very similar to those used by GPs in their diagnostic reasoning: that is, a combination of non-analytic and analytic reasoning [46]. GP clinical supervisors describe the identification of unprofessional behaviour as a process that starts with getting a ‘sense of alarm’. We found three types of such a sense of alarm: a ‘gut feeling’, a ‘loss of enthusiasm for teaching’ and ‘fuss surrounding the resident’. These automatic, fast-emerging feelings of ‘something is wrong with this resident but I cannot figure out what it is yet’ parallels the non-analytic diagnostic reasoning processes [46]. This difficulty to provide detailed and focused descriptions of what is actually wrong reflects the experience that unprofessional behaviour often is difficult to pinpoint [12, 21, 24]. However, GPs are experienced in working with uncertainty: they use their gut feeling and apply the same methods as when gut feeling plays a role in the diagnostic processes of a patient’s complaints [46, 47]. When GP clinical supervisors recognise a sense of alarm, they slow down and ‘switch to analytical reasoning’, in precisely the same way as when gut feeling emerges in medical decision-making [46]. In this analytic process, information is gathered, also using Compass and conversations are planned with other stakeholders: faculty of the day release program are then consulted to collaborate on a remediation plan. When lapses in professionalism persist, the ‘designated professionalism faculty member’ is consulted. In contrast to earlier reports in the medical education literature, we found that identification of unprofessional behaviour and its remediation appear not to be consecutive, but rather intertwined phases [11, 12, 48].

#### Remediation plans

Remediation plans for GP trainees having difficulties are mostly formulated in consultation with faculty. During our interviews, most statements about remediation were made at the level of behaviour and competencies. This can be partly explained by the fact that participants were primed by the terms ‘unprofessional behaviour’ and ‘remediation’, used by the interviewer. Another explanation could be that when we asked participants about remediation, ‘official’ remediation is what came to mind: this is the kind of remediation they felt was only needed to guide residents with a lack of introspection. When introspection is missing, questions addressing deeper issues like beliefs and values, identity and mission usually don’t find fertile soil, because for a discussion on those levels, introspection is an absolute prerequisite [7, 49]. A last explanation could be that in the past two decades competencies have become the dominant language of GP training, making it difficult to work with a richer vocabulary and a more holistic view [50].

## Strengths and limitations

Where previous studies have explored unprofessional behaviours and remediation in the UGME setting, the present study is the first to explore these topics in the PGME setting. The major strengths of our study include its open, exploratory nature and its use of a large, diverse and broadly selected group of participants. Although we deliberately did not provide the participants with definitions of the concepts of professionalism and remediation, the open, exploratory nature of the study can also be seen as a limitation, as the participants may have interpreted these concepts in different ways. Further, we limited this study to Dutch GP training institutes. Although the goal of qualitative studies is not to generalise, having interviewed a diverse group of 55 GP clinical supervisors in eight focus groups from four different Dutch GP training institutes and having interviewed all eight Dutch designated professionalism faculty members, we provide a rich but contextualised understanding of unprofessional behaviours and remediation in a PGME setting. Therefore, we think our study has implications for practice and further research in PGME.

Being interviewed by a colleague (PB) could have negatively affected data collection [31]: the participants might have provided ‘socially desirable’ answers. However, the interviewer kept a research journal in which he reflected on his role in each interview and discussed this in the research group, which added to the rigour of the study. Being interviewed by an intrinsically interested and trustworthy colleague could equally have a positive influence: participants seemed to experience a confidential atmosphere with an interviewer who recognised their daily challenges and thus provided rich ‘inside information’.

## Implications for research and practice

The results of this research provide GP clinical supervisors and faculty with an appropriate language to describe unprofessional behaviours among residents. The descriptors and their categories can facilitate timely identification and thus reduce the problem of ‘failure to fail’ [12, 23]. Further research is needed on the two novel descriptors: ‘Nervous exhaustion complaints’ and ‘Nine-to-five mentality’. Especially the viewpoint of the residents needs to be explored as well as whether the current generation of physicians makes different behavioural choices compared to their older colleagues when certain values are at stake. Moreover, our findings shed light on how GP clinical supervisors and faculty remediate these behaviours as well as on areas of potential improvement and further reduction of the ‘failure to fail’ problem [12, 23]. Firstly, that a ‘sense of alarm’ concerning a resident always deserves to be reflected upon by GP clinical supervisors and faculty. Signs of unprofessional behaviours should be discussed with the resident as early as possible, giving the resident the opportunity to adjust the formation of their professional identity in time. This is especially the case when a lack of introspection is suspected, as introspection is a prerequisite for learning, and improving the capacity for introspection takes time. Secondly, there appears to be scope for GP clinical supervisors to develop their remedial skills, especially when confronted with residents whose introspection is hampered and for whom a the usual PIF approach is insufficient. The comprehensive multi-level professionalism framework might be of help here. This framework can serve to guide the remediation of unprofessional behaviour by encouraging reflection on all important levels that influence professionalism [7]. Furthermore, in faculty development courses on professionalism, attention might be paid to the finding that most GP clinical supervisors suspected a lack of introspection as the underlying problem when informal feedback was not followed by improvements. Supervisors have to be trained in how to distinguish between residents having a problem in introspection, or supervisors themselves are insufficiently aware of their own limitations.

Further research is needed to identify the best way to remediate unprofessional behaviours, especially when introspection is hampered, as well as the suspected beneficial role of ‘deeper’ levels of the professionalism framework [7].

## Conclusions

The results of this study provide appropriate language as well as a better understanding of unprofessional behaviours among GP residents, as well as remediation of those behaviours, according to GP clinical supervisors and faculty. Most unprofessional behaviours parallel the descriptors of the original Four I’s model and can be mapped to the categories of the Four I’s model. The results expand upon the Four I’s model by adding the descriptors: ‘Nervous exhaustion complaints’ and ‘Nine-to-five mentality’. The diagnostic and remediation phases appear non-consecutive and intertwined. Behaviours from the categories Involvement and Interaction receive an informal or ‘pedagogical‘type of remediation. Lack of Introspection or Integrity receives strict remediation on the more quantifiable levels of behaviour and competencies. The findings of this study can assist timely identification and remediation in the future.

## Data Availability

The datasets used and/or analysed during the current study are available from the corresponding author (Pieter C. Barnhoorn, p.c.barnhoorn@lumc.nl) on reasonable request.

## Appendix 1: Interview guide (main questions) individual interviews with designated professionalism faculty members and focus group interviews with GP clinical supervisors

1. What kind of unprofessional behaviour is seen among GP residents?
2. What is the procedure at your faculty if unprofessional behaviour is reported?
3. How is unprofessional behaviour remediated?
4. Do you have examples of success and failure in remediation?
5. Are there other things we need to know on this subjects?

## Abbreviations

GP: general practice
PIF: professional identity formation
PGME: postgraduate medical education
UGME: undergraduate medical education
